# Sample size issues in time series regressions of counts on environmental exposures

**DOI:** 10.1186/s12874-019-0894-6

**Published:** 2020-01-28

**Authors:** Ben G. Armstrong, Antonio Gasparrini, Aurelio Tobias, Francesco Sera

**Affiliations:** 1 Department of Public Health, Environments and Society, London School of Hygiene and Tropical Medicine (LSHTM), 15-17 Tavistock Place, London, WC1H 9SH UK; 2 Centre for Statistical Methodology, London School of Hygiene & Tropical Medicine (LSHTM), Keppel Street, London, WC1E 7HT UK; 3 Institute of Environmental Assessment and Water Research (IDAEA), Spanish Council for Scientific Research (CSIC), C/ Jordi Girona 18-26, 08031 Barcelona, Spain

**Keywords:** Statistics, Sample size, Power, Poisson regression, Time series regression, Environment

## Abstract

**Background:**

Regression analyses of time series of disease counts on environmental determinants are a prominent component of environmental epidemiology. For planning such studies, it can be useful to predict the precision of estimated coefficients and power to detect associations of given magnitude. Existing generic approaches for this have been found somewhat complex to apply and do not easily extend to multiple series studies analysed in two stages. We have sought a simpler approximate approach which can easily extend to multiple series and give insight into factors determining precision.

**Methods:**

We derive approximate expressions for precision and hence power in single and multiple time series studies of counts from basic statistical theory, compare the precision predicted by these with that estimated by analysis in real data from 51 cities of varying size, and illustrate the use of these estimators in a realistic planning scenario.

**Results:**

In single series studies with Poisson outcome distribution, precision and power depend only on the usable variation of exposure (i.e. that conditional on covariates) and the total number of disease events, regardless of how many days those are spread over. In multiple time series (eg multi-city) studies focusing on the meta-analytic mean coefficient, the usable exposure variation and the total number of events (in all series) are again the sole determinants if there is no between-series heterogeneity or within-series overdispersion. With heterogeneity, its extent and the number of series becomes important. For all but the crudest approximation the estimates of standard errors were on average within + 20% of those estimated in full analysis of actual data.

**Conclusions:**

Predicting precision in coefficients from a planned time series study is possible simply and given limited information. The total number of disease events and usable exposure variation are the dominant factors when overdispersion and between-series heterogeneity are low.

## Background

Regression analyses of time series of disease counts on putative environmental determinants, especially air pollution and weather, have been a prominent component of environmental epidemiology of the past quarter century, with no sign of diminishing [1–5]. The units (temporal resolution) are often days, but sometimes weeks, months, or years, and duration can be from a year or less to many decades. For planning such studies, it may be useful to predict the precision of coefficients that will be estimated, or predict power to identify non-null associations. Sometimes, available data is fixed, so that the question (for example for research funders) is whether the new information obtainable from analysing them is worth the cost of doing so, or gives adequate protection against false positives [6]. Sometimes, new data can be obtained at a cost, and a choice needs to be made as to the number of years or the number of locations to collect data from.

Most epidemiologists will understand, as we confirm below, that precision and power will depend on the number of observations (eg days), the total number of disease events or mean events/day, and, for multi-series (eg multi-city) studies, the number of series. The nature of that dependence and whether there are other factors is not generally known and little addressed in the time series regression literature.

The only published method we have found addressing these questions specifically for time series regression of counts focused on power estimation and used simulations [7]. There have in addition been long-published methods for sample size determination and power estimation more generally in generalised linear models [8–10], with some focused on Poisson regression [11]. At least two computer packages have implemented some of these: G*Power, which is free [12], and NCSS:PASS, which is commercially available [13]. However, it has been noted that application to the count time series context was not straightforward [7]. Calculations need to be tailored to the specifics of each study, and the algorithmic nature of the approaches does not facilitate insight into primary determinants of precision and power in typical time series regression contexts. It is also not clear that existing generic methods can extend to multi-series studies analysed in the typical two-stage approach, comprising series-specific regressions then meta-analysis of coefficient estimates.

In this paper we propose some simple approximate formulae for standard error (SE) of the coefficient of interest (and thus precision) requiring only quantities likely to be estimable at the planning stage of a study. This SE formula also allows estimation of width of confidence interval (CI), power to detect an association of specified size, and number of days or number of disease events required for given precision or power. We do not however consider our focus exclusively to be prediction of power, as has been traditionally the case in sample size discussions, following the current trends in epidemiology to reduce emphasis on significance testing [14]. After considering single-series studies we extend the approach to estimate precision in a multi-series study that aim to estimate the mean association (or effect) in a two-stage analysis. We illustrate use of the approximations in a worked planning scenario, and finally evaluate the accuracy of the approximations with existing data sets.

## Methods

### Approximate expressions for precision – single series

#### Basic model and terminology

At time period (usually day) i (1 … n) we have data

*Y*_*i*_ = outcome count (for simplicity we will refer to deaths),

*x*_*i*_ = exposure of interest; without an explicit distributed lag structure other than possibly using a mean over a lag interval as exposure

***z***_***i***_ = vector of additional potentially confounding explanatory variables including those for control of temporal patterns such as spline functions of date. Although many applications use daily series, nothing in our approach requires this. Precision and power for any other temporal resolution (weeks, months, years) can be approached using the same expressions. For simplicity we assume a linear association between the exposure of interest and the logarithm of the outcome counts

$$ E\left({Y}_i\right)=\exp \left(\alpha +\beta {x}_i+\boldsymbol{\gamma} {\boldsymbol{z}}_{\boldsymbol{i}}\right) $$

We will use the term $SE\left(\hat{\beta}\right)$ for standard error of an estimate of the coefficient of interest β and $\mathrm{V}\left(\hat{\beta}\right)$ for its sampling variance. We denote approximate estimators of SE as $S{E}^{\ast}\left(\hat{\beta}\right)$. SD(x) denotes the standard deviation of x. In the main text we state the approximators we propose, and give derivations of them in Additional file 1.

#### Expressions for standard error

The simplest approximation is:

1 $$ S{E}_{Poiss- crude}^{\ast }\ \left(\hat{\beta}\right)=\frac{1}{\sqrt{\varSigma {Y}_i}\times SD(x)} $$

With covariates z, a more accurate approximation is:

2 $$ S{E}_{Poiss}^{\ast }\ \left(\hat{\beta}\right)=\frac{1}{\sqrt{\varSigma {Y}_i}\times SD\left(x|z\right)} $$

Where *SD*(x|**z**) is the standard deviation of the residuals of the exposure of interest in a linear regression of x on **z**, which we term the “usable SD of x”. *SD*(x|**z**) can also be written $SD(x)\times \sqrt{\left(1-{R}_{x\mid z}^2\right)}$, where R^2^_x|z_ is the fraction of variance of x explained by the covariates **z**. The square of the ratio $S{E}_{Poiss- crude}^{\ast }\ \left(\hat{\beta}\right)/S{E}_{Poiss}^{\ast }\ \left(\hat{\beta}\right)= SD(x)/ SD\left(x|z\right)$ is also referred to as the variance inflation ratio [10].

Often residual variance in counts is higher than that of a Poisson distribution by some multiple *φ* (i.e. scale overdispersion). This is called a quasi-Poisson model, and here the approximation (2) becomes

3 $$ S{E}_{Q- Poiss}^{\ast }\ \left(\hat{\beta}\right)=\frac{\sqrt{\varphi }}{\sqrt{\varSigma {Y}_i}\times SD\left(x|z\right)} $$

Again this does not depend on number of days n given *ΣY*_*i*_. In summary, we have proposed a simple approximate formulae for $SE\left(\hat{\beta}\right)$, which depends only on the total number of deaths over the course of the study, usable variation of x, and overdispersion, not on the length of the series n other than through its influence on the total number of deaths *ΣY*_*i*_. We discuss how these determinants might be estimated prior to conducting a study later.

### Approximate expressions for precision – multiple series

We now consider *J* series *j* = 1,…*J*, from each of which we will estimate ${\hat{\beta}}_j\;\mathrm{and}\;V\ \left({\hat{\beta}}_j\right)$, and from these estimate an overall mean ${\hat{\beta}}_{m-a}\;\mathrm{and}\;V\ \left({\hat{\beta}}_{m-a}\right)$. For simplicity we will consider extensions only for the single-series approximators (2) and (3), so that *SD*(x|z) is assumed known, and for tractability we assume that this is constant across studies. We also assume that the series, and hence estimates of effect measures, are independent.

With all series wholly Poisson, the variance of the fixed effects (inverse-variance weighted) meta-analysis mean coefficient can be approximated:

4 $$ S{E}_{F-E}^{\ast }\ \left({\hat{\beta}}_{m-a}\right)=\frac{1}{\sqrt{\sum \limits_{i=1,{n}_j;j=1,J}{Y}_{i,j}}\times SD\left(x|z\right).} $$

Thus, the precision again depends only on the total number of deaths, not additionally on the number of days in the series or the number of series.

With overdispersion *φ* in all the series, this is modified to:

5 $$ S{E}_{F-E}^{\ast }\ \left({\hat{\beta}}_{m-a}\right)=\frac{\sqrt{\varphi }}{\sqrt{\sum \limits_{i=1,{n}_j;j=1,J}{Y}_{i,j}}\times SD\left(x|z\right).} $$

In practice dispersion is unlikely to be the same in all series, but we propose the expression as a simple approximation to series with average dispersion *φ*.

When there is heterogeneity between series estimates (variance τ^2^, comprising a fraction I^2^ of total variance) and analysis with a random effects model, our best approximation is:

6 $$ S{E}_{R-E}^{\ast }\ \left({\hat{\beta}}_{m-a}\right)=\sqrt{\frac{1}{\sum \limits_{j=1,J}\left[{\left\{{\tau}^2+{V}^{\ast }\ \left({\hat{\beta}}_j\right)\right\}}^{-1}\right]}} $$

where ${V}^{\ast }\ \left({\hat{\beta}}_j\right)$ is the estimated variance of ${\hat{\beta}}_j$ in location j using one of the single-series estimates (1)–(3) discussed above, and τ is the underlying heterogeneity SD. However, this requires estimation variance to be predicted separately for each series, as well as τ. For a simpler though more approximate formula we further assume $\mathrm{SE}\ \left({\hat{\beta}}_j\right)$ and hence $\mathrm{V}\ \left({\hat{\beta}}_j\right)$ is constant over locations j. In this case the approximation simplifies to

7 $$ S{E}_{R-E, approx2}^{\ast}\left({\hat{\beta}}_{m-a}\right)=\frac{S{E}_{F-E}^{\ast}\left({\hat{\beta}}_{m-a}\right)}{\sqrt{\left(1-{I}^2\right)}} $$

This has the form of a “heterogeneity corrected” version of one of the approximations of the fixed effects mean, $S{E}_{F-E}^{\ast}\left({\hat{\beta}}_{m-a}\right)$. Other approximators are noted in Additional file 1. It gives insight to consider the unusual case of extreme heterogeneity τ >> $\mathrm{SE}\left({\hat{\beta}}_j\right)$, as this allows a particularly simple approximation:

8 $$ S{E}_{extreme\; het, RE}^{\ast}\left({\hat{\beta}}_{m-a}\right)=\frac{\tau }{\sqrt{J}}, $$

essentially the same as for a simple sample mean. Thus in this case precision depends only on the number of series, and the heterogeneity variance. More generally, we can say qualitatively that as heterogeneity increases relative to expected series-specific coefficient sample variances, the standard error of the overall mean coefficient will depend more on the number of series included and less on the number of deaths, overall or per city.

### Implications for study power

Power of studies to find associations, given a true population hypothesized value β_H1_ for β depends on the standard error, so can be deduced from any of the approximations discussed above:

9 $$ \mathrm{Power}\cong \phi \left[|{\beta}_{H1}|/ SE\left(\hat{\beta}\right)-{z}_{a/2}\right], $$

where α here is the significance level of the test, z_α/2_ is the standard normal deviate defining a right tail probability of α/2 (generally 0.05, with z_α/2_ = 1.96) and *ϕ*( ), is the Gaussian CDF. From this we can also derive the smallest true value of β that can be detected at a given significance level and power:

10 $$ {\beta}_{\mathrm{smallest}\kern0.17em \mathrm{detectable}}\cong \left({z}_{\alpha /2}+{z}_{1- power}\right) SE\left(\hat{\beta}\right) $$

For example, the smallest detectable value of a coefficient at α = 0.05 and power 0.8 is (*z*_0.025_ + *z*_0.2_) =(1.96 + 0.84) = 2.8 times $SE\left(\hat{\beta}\right)$.

And finally, from expression (9)–(10), once one of the approximate expressions for SE(β) is chosen, we can estimate number of deaths required for a given precision or power. In particular using $S{E}_{Poiss}^{\ast }\ \left(\hat{\beta}\right)$ (expression 2):

11 $$ \varSigma {Y}_i\cong {\left[\frac{\left({z}_{\alpha /2}+{z}_{1- power}\right)}{\mid {\beta}_{H1}\mid \times SD\left(x|z\right)}\right]}^2 $$

## Results

### Graphical presentation of application of the expression for power in a range of scenarios

**Fig. 1:**
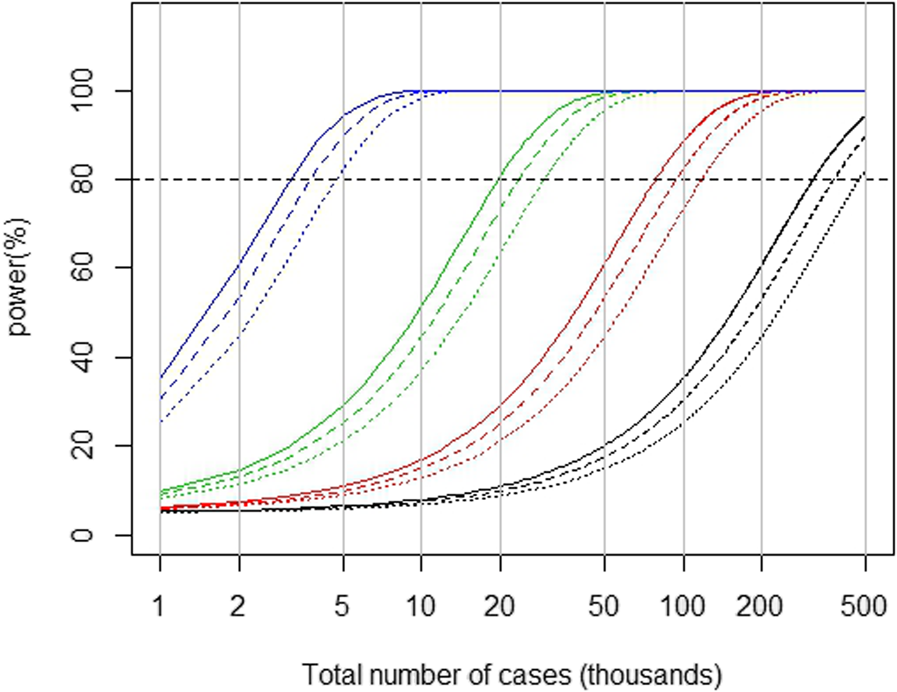
Approximate power as a function of number of cases, size of risk to be detected in relation to usable exposure spread, and overdispersion. Blue lines (left cluster): coefficient (log(RR) per useable SD(x|z)) = 5%. Green lines (middle-left cluster): coefficient = 2% per SD(x|z). Red lines (middle right cluster): coefficient = 1% per SD(x|z). Black lines (right cluster): coefficient = 0.05% per SD(x|z). Within clusters, from left (top): Solid (left): dispersion = 1.0. Dashed (middle): dispersion = 1.2. Dotted (right): dispersion = 1.5

Figure 1 shows some examples of how power depends on number of deaths for a range of values of true underlying coefficients of (log(RR) per unit x) and dispersion. For a condensed presentation we show curves for coefficients per one unit of usable exposure standard deviation. The figure applies to single series studies and also to multi-series studies if a fixed effect analysis is performed and if SD(x|z) and overdispersion is similar across series, as shown above (expressions (4) and (5)). The R-code used to produce this figure, including a general-purpose function for estimating standard error and power (using expressions 3 and 9 above), is included in Additional file 2.

The figure shows that apart from number of deaths power depends most on the strength of the underlying association, the coefficient of log(RR) per SD(x|z). In particular the figure suggests that only if dispersion is substantial (say > 1.2) would consideration of it be important. For a coefficient of 2% per SD(x|z), not unusually a target in environmental studies, about 20,000 deaths would be required to give power of around 80% without overdispersion. That would not change whether it were a 1-year series of about 60 deaths/day or a 10-year series of about 6 deaths/day unless dispersion differed radically. It would also apply to a 10-series study each of 1 year with 6 deaths a day if the series estimates were homogeneous.

### Comparing our approximate power estimate with that estimated by standard power software

We compared the power estimates from our expression the power expression (9) with those obtained from the general purpose program G*Power (Poisson option: details in Additional file 3). For this we used within expression (9) the simple precision approximator in expression (2). We found excellent agreement with the G*Power estimate based on an assumed normally-distributed exposure. For example G*Power estimated 80% power for the example we noted in the previous paragraph, when we input a sample size (number of days) and event rate such that there were 20,000 deaths and a coefficient of 2% per (SD(x|z)). Assuming a uniform distribution of exposure with the same standard deviation also gave power = 80%. Lognormal and exponential distributions showed a very slightly higher power (80.4 and 80.1%), presumably because of their skewness.

### Worked example of using the formulae in planning a study

Here we illustrate how these expressions might help in a planning scenario. Suppose we plan to investigate the association of airborne particulate matter, measured as PM_2.5_ with daily counts of infant deaths in England, considering in the first place London. PM_2.5_ has been measured consistently in England since about 2009. Daily mean PM_2.5_ is available for most major metropolitan areas since about 2009 [15]. To get a rough estimate of the between-day variation (required to predict precision and power) we obtained daily data for one of the London background sites (Bloomsbury), at which from 2009 to 2016 the standard deviation was 9.7 μg/m^3^.

To estimate the variation “usable” in an epidemiological study in which a date spline of 7 degrees of freedom per year would be included to control for seasonality and time trends and indicators for day-of-week, we calculated the standard deviation of residuals (SD_res_) after regressing PM_2.5_ on these terms, which was 8.5 μg/m^3^. A final analysis of the study would likely control also for temperature, and possibly one or more other pollutants, say ozone. This can be done using the same approach as used for seasonality etc., at the cost of the time taken to assemble the data, if available. We did this using public domain data, using a spline of 4 degrees of freedom for temperature (now SD_res_ = 8.1) and linear term for ozone (SD_res_ = 7.6).

The number of infant deaths in London in 2013, approximately in the middle of a likely study period, was 486 [16]. Assuming that at the time the study was conducted data would be available until the end of 2016, total infant deaths over the 8 years 2009–2016 would be about 486X8 = 3888.

By using expression (2), we can then estimate the standard error of regression coefficient (increment in log RR per 10 μg/m^3^ PM_2.5_). If we use the last “fully adjusted” estimate of usable SD (7.6 μg/m^3^), this gives:

$$ S{E}_{Poiss}^{\ast }\ \left(\hat{\beta}\right)=\frac{1}{\sqrt{\left\{\varSigma {Y}_i\right\}}\times SD\left(x|\mathbf{z}\right)}=\frac{1}{\sqrt{3888}\times 0.76}=0.021 $$

This estimate assumes no overdispersion, an assumption we will argue later (see Discussion) is reasonable for this context.. In this case, using the simple unconditional standard deviation of PM_2.5_ (9.7 μg/m^3^) would only slightly exaggerate precision ($S{E}_{Poiss- crude}^{\ast }\ \left(\hat{\beta}\right)=0.016$), reflecting the modest seasonality and trend in PM_2.5_. From $S{E}_{Poiss}^{\ast }\ \left(\hat{\beta}\right)$ we can deduce the width of CI (2X1.96*0.021 = 0.082). A coefficient of 0.06 was found in one recent study) (Yorifuji et al. 2016). Power (using expression (9)) would be good (82%) to detect an underlying coefficient of this size, but would be lower to detect the smaller coefficients found in some other studies [17]. The smallest positive coefficient detectable with 80% power would be 2.8X0.021 = 0.059.

To answer questions such as “what length of series in London would be required to obtain a specific power?” we could use expression (11). For example for 90% power (hence z_(1–0.9)_ = 1.28) this estimates the require number of deaths to detect a coefficient of 0.06 as $\varSigma {Y}_i\cong {\left[\frac{\left(1.96+1.28\right)}{0.06\times 0.76}\right]}^2=5048$. If we continue to assume 486 deaths per year, the series would thus need to have 5048/486 = 10.4, i.e., about 10 years.

Alternatively we might seek to improve precision, power, or detectable effect by using multiple series. Including nine other English major metropolitan areas was found in a previous study to increase the number of infant deaths by 2.5 fold [18], so such a study might have 2.5 X 3888 = 9720 deaths, which in the absence of between-area heterogeneity and if the usable variation in PM_2.5_ was similar in other areas would reduce the standard error to 0.013, and the smallest detectable value to 0.037. If heterogeneity was expected the task is more complex and the prediction less certain. With predicted τ and individual series SE(${\hat{\beta}}_j$) values one could use expression (6). An I^2^ value might more usually be reported in comparable published studies than a τ value which if we also ignore variation in SE(${\hat{\beta}}_j$) values would allow us to use expression (7) to find how much the fixed effect meta-analytic mean SE of 0.013 would be increased. For example, for I^2^ values of 10,25,50, and 75% would increase the fixed effect SE by 0.5,3,15, and 51% respectively (multiple = 1/√((1-I^2^)).

### Comparison of the above approximations with conventional (quasi) Poisson estimates from real data

We tested the accuracy of the approximations (1)–(8) for $\mathrm{SE}\left(\hat{\beta}\right)$ with the actual values estimated from daily temperature and mortality data from 51 provincial capitals of Spain 1990–2010, previously used in several published studies [19, 20] and summarised in Additional file 4. Specifically we considered a simple model with all natural deaths as the outcome and as explanatory term of interest (x) a linear model for “heat” defined as (lag 0) temperature above the 75th centile of daily mean temperature in that city. The covariates considered were a natural spline function of time with 7df per year, and indicators for day of week.

When true values for the expression “input” parameters SD(x), SD(x|z) and overdispersion were assumed known, thus evaluating approximations in the expressions themselves, only the crudest approximation (1; ignoring covariate impact on exposure variation) gave error that is non-negligible in this context (Table 1A and detail in Additional file 4A). This is due to its ignoring the strong seasonality of the exposure considered (heat) (R^2^_x|z_ = 0.4 on average). For other exposures with less strong association with covariates this approximation would perform better. Allowing for overdispersion (3) gave little improvement in general in these data, where dispersion was on average 1.03 and never exceeded 1.15. However, the poorest performance of the basic Poisson approximation (2) (underestimating $SE\left(\hat{\beta}\right)$ by 11 and 10%) was in the cities with relatively large overdispersion in these cities (1.15 and 1.14). These two cities had the largest daily mean deaths (48 and 75), and in general overdispersion was strongly associated with mean deaths (Additional file 4: Figure A4.2).

**Table 1:** Performance of approximations of precision of approximations to $SE\left(\hat{\beta}\right)$ in 51 cities

Estimator (text expression number)	Distribution of % error in estimators of $SE\left(\hat{\beta}\right)$ over the 51 cities			
	Mean (bias)	Mean absolute	Lowest	Highest
A: Using known exposure distribution				
Poisson crude (1)	− 39.3	39.3	− 58.0	−19.7
Poisson (2)	−3.8	3.9	−11.5	2.6
Q-Poisson (3)	−2.3	2.5	−5.0	4.0
B: Using exposure distribution from year 1				
Poisson_crude (1)	−37.1	37.1	−58.6	−18.3
Poisson (2)	7.1	15.7	−28.9	44.5
Q-Poisson (3)	9.1	17.1	− 32.4	46.7

The table summarises the distribution of errors in three approximations to the standard error of the coefficient of heat ($SE\left(\hat{\beta}\right)$) in the 51 Spanish provincial capitals

% error = 100*[approximation-(true value)]/(true value)]

We then used first year data alone to approximate SD(x), SD(x|z) and overdispersion, to mimic the sort of proxy data that might be used in real study planning (Table 1B and Additional file 4B). Here error frequently was more than ±10%, even in the best approximators.

Table 2 shows performance of approximators (4)–(7) for the standard error of the multi-series meta-analytic mean of the 51 coefficients. Here there was clear heterogeneity (I^2^ = 65.3%, τ = 0.0055), so all estimates of SE ignoring this (whether using actual data or a prior approximation) underestimated the more realistic true random effects SE. However, the approximate estimators estimated the fixed effect SE well. If the extent of heterogeneity (τ) could be accurately predicted, the approximate expressions for the random effects SE performed well. Estimation of SE when I^2^ could be predicted, assuming SE(${\hat{\beta}}_j$) constant (7) led to moderate underestimation of SE, probably because the actual SE(${\hat{\beta}}_j$) varied considerably. The underestimation of SE by 25% assuming extreme heterogeneity (8) strikes a cautionary note, that the actual I^2^ of 65% is not extreme enough for this estimate to be for this approximation to be close.

**Table 2:** Performance of approximations of $\mathrm{SE}\left({\hat{\beta}}_{m-a}\right)$ $SE\left(\hat{\beta_{m-a}}\right)$ of meta-analytic mean coefficient over 51 cities ($\hat{\beta}$ =2.19%)

Estimator (text expression number)	Estimated $\mathrm{SE}\left({\hat{\beta}}_{m-a}\right)$ (%)	% error w.r.t. actual FE	% error w.r.t. actual RE
Fixed effect model			
Actual $S{E}_{F-E}\ \left({\hat{\beta}}_{m-a}\right)$	0.054	0.0	− 46.7
Poisson (4)	0.050	−7.6	−50.8
Q-Poisson (5)	0.051	−6.2	−50.0
Random effects model			
Actual $S{E}_{R-E}\ \left({\hat{\beta}}_{m-a}\right)$	0.102	87.6	0.0
τ and $S{E}_{Poiss}^{\ast }\ \left({\hat{\beta}}_j\right)$ known (6)	0.101	NA	−1.4
I^2^ and $S{E}_{Poiss,F-E}^{\ast}\left({\hat{\beta}}_{m-a}\right)$ known (7)	0.085	NA	−16.4
extreme heterogeneity assumed (8)	0.076	NA	−25.3

*RE* Random effects, *FE* Fixed effects, *w.r.t.* With respect to

The first column shows the standard error of the meta-analytic mean, estimated by each method. The remaining two columns show the % error in each estimate of SE(β ^) with respect to gold standards actual FE estimate (column 2) and actual RE estimate (column 3) = 100*[approximation-(true value)]/(true value)]

## Discussion

We have provided simple approximate estimators for precision of estimates of coefficients of interest and hence power that can be helpful in advance of undertaking a study. The simplicity of the estimators allow some general guidelines to be identified: For single series, the total number of deaths and the useable variation (SD) of exposure are the dominant factors unless overdispersion is severe. For multiple series from which a meta-analytic mean is to be estimated, the aggregate of these factors over series remain dominant if there is little underlying heterogeneity of coefficients between series, but if there is such heterogeneity its extent and the number of series become important.

Our application of the estimators for single series to examples in which we in fact knew the precision realised, and comparison of power estimates to those from algorithms implemented in the package G*power suggested that the approximations we made were good to the extent that total number of deaths and usable exposure variation could be predicted. For meta-analytic means of multiple series estimates were less reliable, in particular if heterogeneity was present and not allowed for.

The finding that precision and power are dominated by total number of events is consistent with but goes further than the power simulations published previously [7], where it was observed that “increasing time-series length and average daily outcome counts both increased power to a similar extent”. This publication also commented: “reduction in power […] can accompany use of multipollutant models”. Although we do not address multi-pollutant models explicitly in this paper, when interest in is effect of each pollutant adjusting for that of others, those other pollutants can be considered covariates in our formulation. Because inclusion of such additional covariates reduces usable exposure SD of the exposure of interest, this statement is also consistent with our result.

The conclusion that the length of the series only influences precision through total event count may seem surprising to some. It does have limitations, but we believe they are minor ones. Some exposures may have larger exposure variation in longer series, but because time series regressions routinely include covariates to model longer-term variations, such as trend or seasonality, the usable standard deviation often changes little. For example SD(x|z) of heat exposure was on average 1.07 over the full 21 years, and 1.06 over just 1 year (for convenience the first). However, aspects other than precision, such as robustness against bias, may diminish in short series. For an extreme example an estimate from a “series” of 10 days with 1000 deaths/day on average may have the same precision as one from a series of 1000 days with 10 deaths/day on average, but would seem more subject to bias, for example some other risk factor happening to be concurrent with the most exposed day or two.

There is an impact of temporal resolution of the series (days, weeks, etc) on precision of coefficients of interest that is not directly evident in our expressions. The usable variability of exposure, which does influence precision, often changes with temporal resolution. Overdispersion of the outcome may also change, in our experience often increasing with longer durations. And there may be epidemiological considerations, for example fine resolution (eg days) is optimal to estimate acute exposure effects, whereas coarser resolution (eg years) has advantages for longer term effects.

There are alternatives to the approach we have proposed. If all counts are large enough to assume that they or their logarithm are normally distributed, and vary only modestly, simpler methods might be used. Otherwise, as we have illustrated, programs such as G*Power can be used for a single series with Poisson deaths to find power given number of days, baseline death rate per day, standard deviation of exposure and variance inflation factor. But this misses the insights of the simpler approximate expressions, in particular that number of days and baseline deaths rate influence precisions and power only though the total number of deaths. If actual linked outcome and exposure data exists, one can simply run the regression to find the precision and hence deduce power. But our approach does not need such detail, and can with limited estimated summaries be used to illustrate, for example, how choices of different cities or length of series lead to different precision and power, allowing better informed design decisions.

A problem with predicting precision and power with any method is that these depend on parameters that are unknown at planning stage, in particular mean counts, usable exposure variation, and overdispersion. Our worked example illustrated some approaches to this such as making estimates from other studies or preliminary data. The Spanish multi-city comparison also suggested that in similar contexts overdispersion would be low (< 1.1) if mean counts were below about 40. Unfortunately few studies report overdispersion quantitatively, but it is our experience that at least for deaths or hospitalisations due primarily to non-infectious diseases this pattern is common. This was one reason we considered the estimate we made for the infant deaths example need not assume overdispersion. However, there may of course be exceptions, and each context should be considered on its merits.

The issue of not knowing the parameters needed to estimate precision and power is particularly acute for multiple series, where as our Spanish cities example illustrated, the extent of heterogeneity in effect estimates over series can be critical. Other simplifying assumptions also were needed, for example all approximations assumed that the usable exposure variation was constant across all studies, expression (5) assumed constant overdispersion, and expression (7) constant SE(${\hat{\beta}}_j$), Uncertainty in approximations will increase with uncertainty in assumptions. It may be useful if such uncertainty is great to make estimates of precision of the desired effect measure under varying assumptions (so possibly using different approximations) as a sensitivity analysis.

There are several limitations in the approach we present. First, our estimators of precision are approximate, and we have not undertaken a comprehensive evaluation of them by simulation. However, the evaluation from real data suggests that primary source of error is not in the expressions but in the limited precision with which input parameters, in particular usable predictor exposure SD, can be predicted at planning stage. This is an issue whatever the accuracy of algorithms to find precision and power for given input parameters. Second, we do not discuss non-linear or distributed lag models. Both of these could be addressed to some extent by estimating precision and power for a simplified model. For example the analysis of Spanish data above approximated the curved linear temperature-mortality curve with a linear-threshold model. To approximate using a distributed lag model to estimate a cumulative risk of all lags we could estimate the usable standard deviation of the running mean of temperature over the most important lags, for example a three-day running mean for heat.

Also, we have not addressed auto-correlation in residuals. If allowed for in the model this would somewhat diminish precision, but since it has usually been found small if present at all, the impact of this is not generally expected to be large. Neither have we considered distributional models other than Poisson with scale overdispersion, for example negative binomial or zero-inflated Poisson. Finally, we do not discuss case cross-over analysis. However, given the equivalence between fixed stratum versions of this approach and time series regression with stepped time functions [21], there seems no reason to believe that the results presented here would not apply provided that the “usable exposure SD” is that conditional on time stratum as well as any other covariates.

## Conclusions

Predicting precision in coefficients from a planned time series study and hence power and detectable effect is possible simply and given limited information. The total number of disease events and usable exposure variation are the dominant factors when overdispersion and for multiple series between-series heterogeneity are low.

Conducting a study without enough power to detect coefficients of plausible magnitude compromises its informativeness and increases the chance of false positives [6]. Coefficients of mortality on air pollution are frequently particularly small in relation to exposure SD, and studies thus require large numbers of deaths, often more than available from a single series. Temperature-mortality coefficients can be somewhat larger, so studies with fewer deaths can still be informative. These expressions for precision and power can allow researchers to plan size more confidently.

## Supplementary information

**Additional file 1.** Derivations of approximations to standard errors of coefficients and smallest detectable coefficient.

**Additional file 2.** R code for power calculation.

**Additional file 3.** Illustration of the use of G*Power.

**Additional file 4.** Details of comparison of estimators and related data in 51 Spanish cities (Figures).

## Data Availability

R-code is provided in Additional file 2. The Spanish daily mortality data was provided by the Spanish National Institute of Statistics (reference PB242/2016), with permission to use for analysis but not to make available to others. The mortality data can be obtained from the Spanish National Statistics Institute.

## Abbreviations

CI: Confidence interval
SD: Standard deviation
SE: Standard error
SE*: Approximate estimate of SE
V: Variance
