# Supplementary material for: Sample size issues in time series regressions of counts on environmental exposures
Source: BMC Med Res Methodol. 2020 Jan 28;20:15. doi: 10.1186/s12874-019-0894-6 (PMC6988321; doi:10.1186/s12874-019-0894-6)
Supplement: Supplementary file 1 — Additional file 1. Derivations of approximations to standard errors of coefficients and smallest detectable coefficient. [file 12874_2019_894_MOESM1_ESM.docx]

# Sample size issues in time series regression studies.

# Additional file 1: Derivations of approximations to standard errors of coefficients and smallest detectable coefficient

## A Single series

Poisson model

To simplify cross-referencing of these derivations of the approximations to their statement in the main text, we use the same expression names and numbers, on the right hand side of the expressions.

Y_i_  ~ Poisson

Then, if y_i_ = ln(Y_i_) is calculable (Y_i_>0), the usual model can be re-cast:

$E\left( Y_{i} \right)=exp(\alpha+ \beta x_{i}+ \boldsymbol{\gamma}\boldsymbol{z}_{\boldsymbol{i}})$ with V(y_i_|x_i_,z_i_)≈1/E(Y_i_)

Further approximating (Y_i_) ≈ $\bar{Y}$, V(y_i_|x_i_,z_i_)≈1/$\bar{Y}$.

This allows use of the standard expression for var($\hat{\beta})$ in a simple regression model:

$$\text{V(}\hat{\beta})\approx\frac{V(y|x)}{n.V(x)}\approx\frac{1}{\bar{Y}.n.V(x)}=\frac{1}{\{\Sigma Y_{i}\}.V(x)}\text{=V}_{Poiss-crude}^{*}\text{ (}\hat{\beta})$$

Or $SE_{Poiss-crude}^{*}\text{ (}\hat{\beta})=\frac{1}{\sqrt{\Sigma Y_{i}}\times SD(x)}$ (1)

Extending to a model with covariates (vector **z,** $E(Y_{i})=\exp\{\alpha+\beta x_{i}+\mathbf{\gamma}^{T}\mathbf{z}_{i}\}$ ), the above holds to the last line, which becomes [need ref– standard LM text?]:

$$\text{V(}\hat{\beta})\approx\frac{V(y|x,\mathbf{z})}{n.V(x).(1-R_{x|\mathbf{z}}^{2})}\approx\frac{V(y|x,\mathbf{z})}{n.V(x|\mathbf{z})}\approx\frac{1}{\bar{Y}.n.V(x|\mathbf{z})}=\frac{1}{\{\Sigma Y_{i}\}.V(x|\mathbf{z})}\text{=V}_{Poiss}^{*}\text{ (}\hat{\beta})\text{ }$$

Or $SE_{Poiss}^{*}\text{ (}\hat{\beta})=\frac{1}{\sqrt{\Sigma Y_{i}}\times SD(x|z)}$ (2)

Where var(x|**z**) is the variance of the residuals of the exposure of interest in a linear regression of x on **z**, which we term the “usable variance of x” (which can also be written var(x)(1-R^2^_x|z_).

Quasi-Poisson model

When residual variance is higher than that of a Poisson distribution by some multiple (i.e. scale overdispersion as in the quasi-Poisson model, then

and

Or (3)

## B: Multiple series

We now consider J series j=1,…J, from each of which we will estimate $\hat{\beta}_{j} \text{and} V\text{(}\hat{\beta}_{j})$, and from these estimate an overall mean $\hat{\beta}_{m-a} \text{and} V\text{(}\hat{\beta}_{m-a})$. Standard theory for meta-analysis shows, for fixed effects (inverse-variance weighted) meta-analysis:

$$V_{F-E}\text{(}\hat{\beta}_{m-a})=\frac{1}{\sum_{j=1,J} \text{\{V(}\hat{\beta}_{j})\}^{-1}}$$

For wholly Poisson series, if we use the previously derived approximation for $\text{V (}\hat{\beta}_{j})$ in the above expression we get:

$$V_{F-E}\text{(}\hat{\beta}_{m-a})\approx\frac{1}{\sum_{j=1,J} \left[V_{j}(x|z)\times\sum_{i=1,n_{j}} Y_{i,j}\right]}$$

As it is usually rare to be able to predict V(x|z) at planning stage it is useful to further approximate by assuming that $V(x|z)$ is constant across studies.

$$V_{F-E}\text{(}\hat{\beta}_{m-a})\approx\frac{1}{\sum_{j=1,J} \left[V(x|z)\times\sum_{i=1,n_{j}} Y_{i,j}\right]}=\frac{1}{V(x|z)\times\sum_{i=1,n_{j};j=1,J} Y_{i,j}}=V_{Poiss,F-E}^{*}\text{(}\hat{\beta}_{m-a})$$

Or $SE_{F-E}^{*}\text{ (}\hat{\beta}_{m-a})=\frac{1}{\sqrt{\sum_{i=1,n_{j};j=1,J} Y_{i,j}}\times SD(x|z).}$ (4)

With overdispersion in all the series, this is modified to:

(5)

We also propose this as an approximation to a set of series with average dispersion .

With heterogeneity between series estimates (variance τ^2^), analysed with a random effects model,

$$\text{V}_{R-E}\text{(}\hat{\beta}_{m-a})=\frac{1}{\sum_{j=1,J} \left[\text{\{}\tau^{2}+V\text{ (}\hat{\beta}_{j})\}^{-1}\right]}\approx\frac{1}{\sum_{j=1,J} \left[\text{\{}\tau^{2}+V^{*}\text{ (}\hat{\beta}_{j})\}^{-1}\right]}=V_{R-E}^{*}\text{(}\hat{\beta}_{m-a})$$

Or $SE_{R-E}^{*}\text{ (}\hat{\beta}_{m-a})=\sqrt{\frac{1}{\sum_{j=1,J} \left[\text{\{}\tau^{2}+V^{*}\text{ (}\hat{\beta}_{j})\}^{-1}\right]}}$ (6)

Where $V^{*}\text{ (}\hat{\beta}_{j})$ is one of the approximations to $\text{V(}\hat{\beta}_{j})$, $V_{Poiss}^{*}\text{ (}\hat{\beta}_{j})$ or $V_{Q-Poiss}^{*}\text{ (}\hat{\beta}_{j})$, giving $V_{Poiss,R-E}^{*}\text{(}\hat{\beta}_{m-a})$ or $V_{Q-Poiss,R-E}^{*}\text{(}\hat{\beta}_{m-a})$ respectively.

If we again further assume V(β_j_) is constant over j (say V(β_all_)), then this simplifies to several alternative expressions, each suggesting other approximations to $\text{V(}\hat{\beta}_{m-a})$:

$SE_{R-E,approx2}^{*}\text{(}\hat{\beta}_{m-a})=\frac{SE_{F-E}^{*}\text{(}\hat{\beta}_{m-a})}{\sqrt{(1-I^{2})}}$ (7)

$SE_{R-E,approx3}^{*}\text{(}\hat{\beta}_{m-a})=\frac{SE*(\beta_{all})}{\sqrt{J(1-I^{2})}}$ (7a)

$SE_{R-E,approx4}^{*}\text{(}\hat{\beta}_{m-a})=\sqrt{\frac{(\tau^{2}+ V*(\beta_{all}))}{J}}$ (7b)

We believe that the first of these is the most directly useful, but the others give insights. In particular

consider the case of extreme heterogeneity τ^2^ >>$\text{V(}\beta_{all})$. Then, from (7b):

$SE_{extreme het,RE}^{*}\text{(}\hat{\beta}_{m-a})=\frac{\tau}{\sqrt{J}}$, (8)

as for a simple mean. Here, for fixed heterogeneity variance, precision depends only on the number of series, and the heterogeneity variance.

## C: Power and smallest detectable coefficient

Power of the to test $H_{1}:\beta=\beta_{H1} \neq0$ against $H_{0}:\beta=0$, is a function of $SE\left( \beta\right)$, and coefficient β. The power function for a two-sided *Z* test (assuming $\beta_{H1}$ positive) is given by

Power = $\phi\left[ {\beta_{H1}}/{SE\left( \beta\right)-z_{\alpha/2}} \right]+\phi\left[ -z_{\alpha/2}-{\beta_{H1}}/{SE\left( \beta\right)} \right]$ , (9a)

where α here is the significance level of the test, z_α/2_ is the standard normal deviate defining a right tail probability of α/2 (generally 0.05, so z_α/2_= 1.96) and $\phi( )$, is the Gaussian cumulative distribution function. As the second term is in most practical contexts (if power>10%) very small, to a good approximation this is

Power $\cong\phi\left[ {\beta_{H1}}/{SE\left( \hat{\beta} \right)-z_{\alpha/2}} \right]$ (9)

Thus given an approximation SE*( $\beta$), we can approximate also the power to detect an hypothesized value of $\beta$.

Changing the subject of this expression to β, the smallest coefficient detectable at a specified power is:

$\beta_{\text{smallest} \text{detectable}}=(z_{\alpha/2}+z_{power})SE(\beta)$ (10)

For example, the smallest detectable value of a coefficient at p<0.05 and power 0.8 is $(z_{0.025}+z_{0.2})$=(1.96+0.84) = 2.8 times SE(β).

And finally, from expression (9)-(10), once one of the approximate expressions for SE(β) are chosen, we can estimate number of deaths required for a given precision or power. For example using $SE_{Poiss}^{*}\text{ (}\hat{\beta})$ (expression 2) and modifying expression (10):

$\begin{matrix} \left|\beta_{H1}\right|=\frac{(z_{\alpha/2}+z_{power})}{\sqrt{\Sigma Y_{i}}\times SD(x|z)} \\ \text{So} \Sigma Y_{i}=\left[\frac{(z_{\alpha/2}+z_{power})}{\left|\beta_{H1}\right|\times SD(x|z)}\right]^{2} \end{matrix}$ (11)
