# Supplementary material for: Sample size issues in time series regressions of counts on environmental exposures
Source: BMC Med Res Methodol. 2020 Jan 28;20:15. doi: 10.1186/s12874-019-0894-6 (PMC6988321; doi:10.1186/s12874-019-0894-6)
Supplement: Supplementary file 2 — Additional file 2. R code for power calculation. [file 12874_2019_894_MOESM2_ESM.docx]

# Sample size issues in time series regression studies.

# Additional file 2: R code for power calculation

# FUNCTION TO ESTIMATE SE(BETA) AND POWER GIVEN N OF CASES (CAN BE A VECTOR),

# HYPOTHESIZED COEFFICIENT, USABLE SDX, OVERDISPERSION, AND TEST SIZE

power.tsr <- function(ncases,usablesdx=1,coef,overdispersion=1,alpha=0.05) {

se<-sqrt(overdispersion)/(sqrt(ncases)*usablesdx)

zetaalpha2 <- qnorm(1-alpha/2)

cum1<-1-pnorm(zetaalpha2+(coef/se))

cum2<-pnorm(coef/se-zetaalpha2)

power<-cum1+cum2

results <- cbind(ncases,se, 100*power)

colnames(results) <- c("ncases","SE","Power%")

fixedparameters <- c(usablesdx,coef,overdispersion,alpha)

names(fixedparameters) <- c("usablesdx","coef","overdispersion","alpha")

output <- list(fixedparameters,results)

names(output) <- c("fixedparamters","results")

return(output) # list of results (matrix) and fixed input parameters (vector)

}

#### CALCULATE POWER CURVES FOR FIGURE 2

n1000cases <- seq(1,500)

coefs <- c(0.005, 0.01,0.02,0.05)

overdisps <- c(1,1.2,1.5)

plot(x=n1000cases, y=115*seq(1,max(n1000cases))/max(n1000cases) ,

type="n", ylab="power(%)", xlab="Total number of cases (thousands)", log="x")

for(coefn in seq(1,length(coefs ))) {

for(overdispn in seq(1,length(overdisps)) ) {

power<- power.tsr(ncases=n1000cases*1000,overdispersion=overdisps[overdispn],

coef=coefs[coefn])$results[,3]

lines(x=n1000cases, y=power, lty=overdispn, col=coefn)

}

}

abline(h=80,lty=2)

abline(v=c(1,2,5,10,20,50,100,200,500), lwd=0.05, col="grey")

### PLANNING SCENARIO:

# LONDON ONLY

(power1 <- power.tsr(ncases=3888 , usablesdx=0.82,coef=.06 ) )

# VERIFY MINIMUM DETECTABLE COEFFICIENT GIVES POWER = 80%

(MDC <- 2.8* power1$results[,"SE"] )

power.tsr(ncases=3888 , usablesdx=0.82, coef=MDC)

# 10 MAJOR METROPOLITAN AREAS (also verifying MDC)

power.tsr(ncases=9720 , usablesdx=0.82,coef=.034 )
