# Supplementary material for: Sample size issues in time series regressions of counts on environmental exposures
Source: BMC Med Res Methodol. 2020 Jan 28;20:15. doi: 10.1186/s12874-019-0894-6 (PMC6988321; doi:10.1186/s12874-019-0894-6)

# Sample size issues in time series regression studies.

# Additional file 3 Illustration of the use of G*Power

The screenshot below shows the application of G*Power to find power for the example highlighted in the main text: number of deaths needed to identify an effect size 2% per SD(x|z) with power 80% and p<0.05. Note that by setting “base rate” (deaths per day) to 1, the “sample size” (number of days) also specifies the number of deaths. Other base rates and sample sizes with other values with the same product, for example 100 and 200, give the same power.


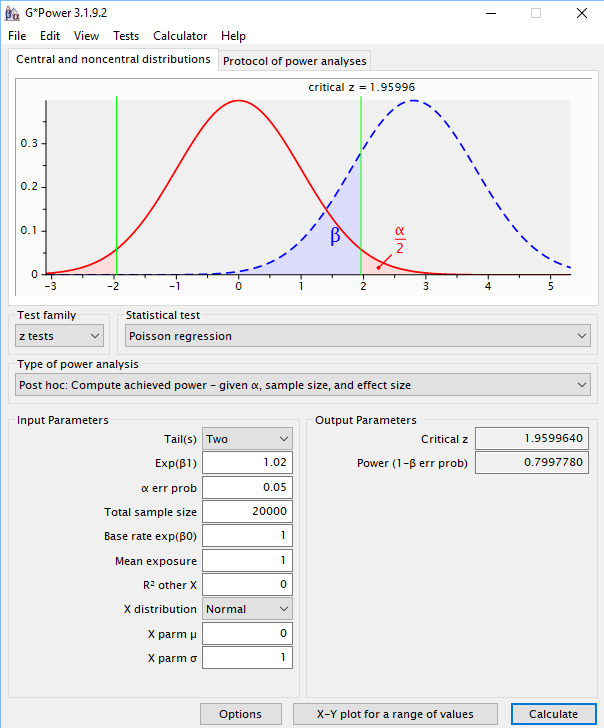

Supplement: Supplementary file 3 — Additional file 3. Illustration of the use of G*Power. [file 12874_2019_894_MOESM3_ESM.docx]
