# Supplementary material for: Sample size issues in time series regressions of counts on environmental exposures
Source: BMC Med Res Methodol. 2020 Jan 28;20:15. doi: 10.1186/s12874-019-0894-6 (PMC6988321; doi:10.1186/s12874-019-0894-6)

# Sample size issues in time series regression studies.

# Additional file 4 Details of comparison of estimators and related data in 51 Spanish cities.

### Details of data:

We collected data from the 51 capital cities between 1st of January 1990 and 31st of December 2010. Daily mortality, obtained from Spain National Institute of Statistics, is represented by counts of deaths for all causes. Mean daily temperature (in ˚C), computed as the 24-hour average based on hourly measurements, was obtained from Spain National Meteorology Agency. A single weather station, located within the urban area or at the near airport, was selected for each city. Single-day missing values were imputed as the average of the days before and after. For periods longer than two days no imputation was done. In total, missing data amount for 0.00% and 2.00% of the mortality and temperature series, respectively.

### Figure A4.1: Errors in approximations to SE(β)


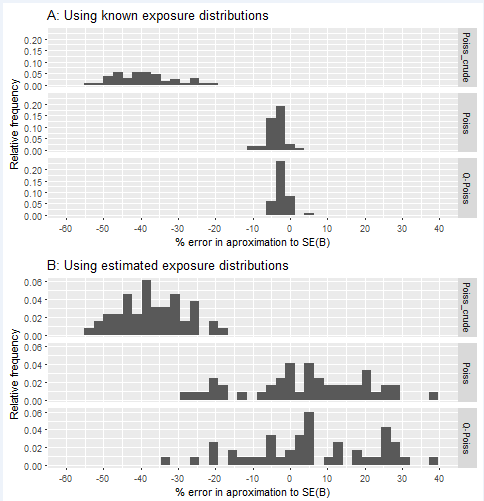


### Figure A4.2: Association of overdispersion with deaths/day.


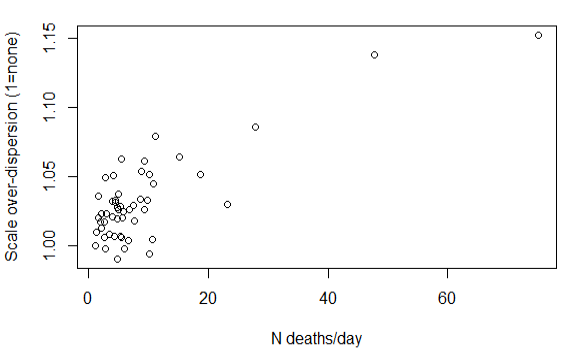

Supplement: Supplementary file 4 — Additional file 4. Details of comparison of estimators and related data in 51 Spanish cities (Figures). [file 12874_2019_894_MOESM4_ESM.docx]
